# A new classification and its value evaluation for intermediate column fractures of the distal radius

**DOI:** 10.1186/s13018-018-0925-8

**Published:** 2018-09-03

**Authors:** Ying Yang, Qudong Yin, Dongchen Li, Yongjun Rui, Yongwei Wu, Yanping Ding, Yan Jiang

**Affiliations:** 1 Department of Radiology, Wuxi the Ninth People’s Hospital Affiliated to Suzhou University, Wuxi, 214062 China; 2 Department of Orthopaedics, Wuxi the Ninth People’s Hospital Affiliated to Suzhou University, Wuxi, 214062 China

**Keywords:** Distal radius fracture, Intermediate column, Classification

## Abstract

**Background:**

Intermediate column fractures of the distal radius (ICF) are fractures only or mainly limited to the lunate fossa of the distal radius. There are no classification systems and its value evaluation for ICF in the literature.

**Methods:**

According to the characteristics of ICF, ICF were divided into the volar, dorsal, split, collapse, and collapse with split types. Inter- and intra-observer agreements were analyzed with kappa statistics. Seventy-four patients with ICF were retrospectively studied from January 2008 to June 2016. Surgical approach and reduction-fixation method were taken under the guidelines of the classification in 54 patients with displaced fractures, while conservative treatment was taken in 16 patients with non-obvious displaced fractures and 4 patients with displaced fractures who declined surgery.

**Results:**

The inter- and intra-observer kappa coefficients were 0.875~0.925 and 0.900~0.950, respectively. All patients were followed up for 13~36 months (average, 18.4 months). At the last follow-up, according to Gartland and Werley score of the functional recovery of wrist, all except 3 patients had excellent or good results (the excellent and good rate was 95.95%).

**Conclusion:**

The classification reflects the characteristics of ICF and may provide an important reference for choosing treatment and evaluating prognosis.

## Background

In 1996, Rikli and Regazzoni [1] presented the three-column theory of fractures of the distal radius, in which the ulnar half of the distal radius, composed of the lunate fossa and the sigmoid notch, was called the intermediate column. The intermediate column is the primary load-bearing and load transfer structure in the wrist, and it plays a key role in distal radius fractures [2–5]. Intermediate column fractures (ICF) of the distal radius are fractures only or mainly limited to the middle column of the distal radius that is a result of an axial load passed to the lunate fossa through the lunate. Some authors used the term die-punch fractures to refer to ICF [6–8]; however, the definition of die-punch fractures has been controversial [9–12], therefore which should be used as less as possible so as to avoid misunderstanding. To our knowledge, no previous study in literature has referred to classification and its value evaluation for ICF. The purpose of this study was to introduce a new classification for ICF and evaluate the value of the new classification.

## Methods

### Classification system

**Fig. 1:**
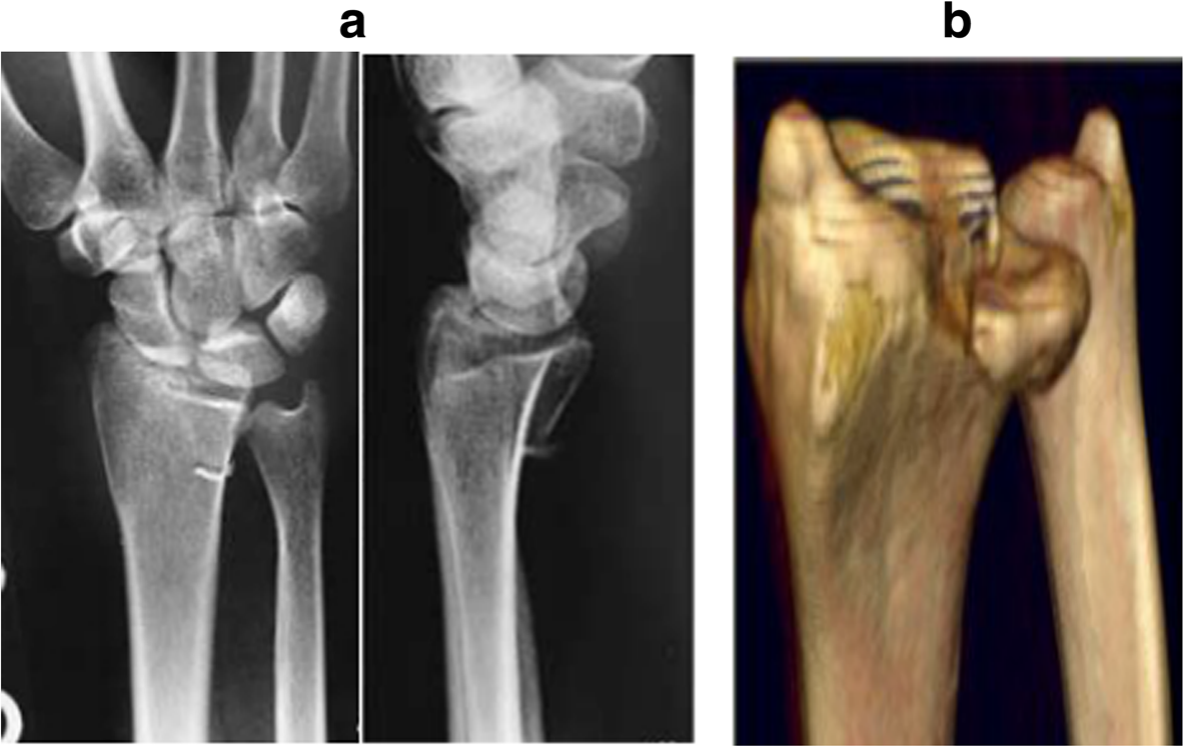
**a**, **b** Volar type of ICF

**Fig. 2:**
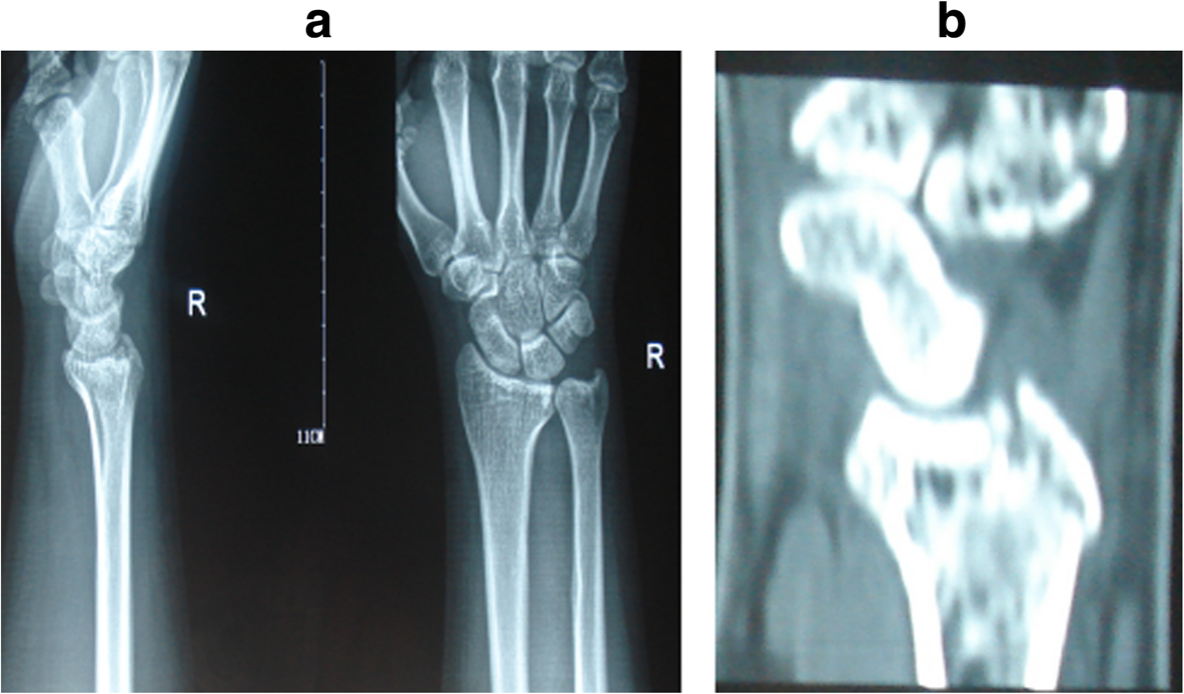
**a**, **b** Dorsal type of ICF

**Fig. 3:**
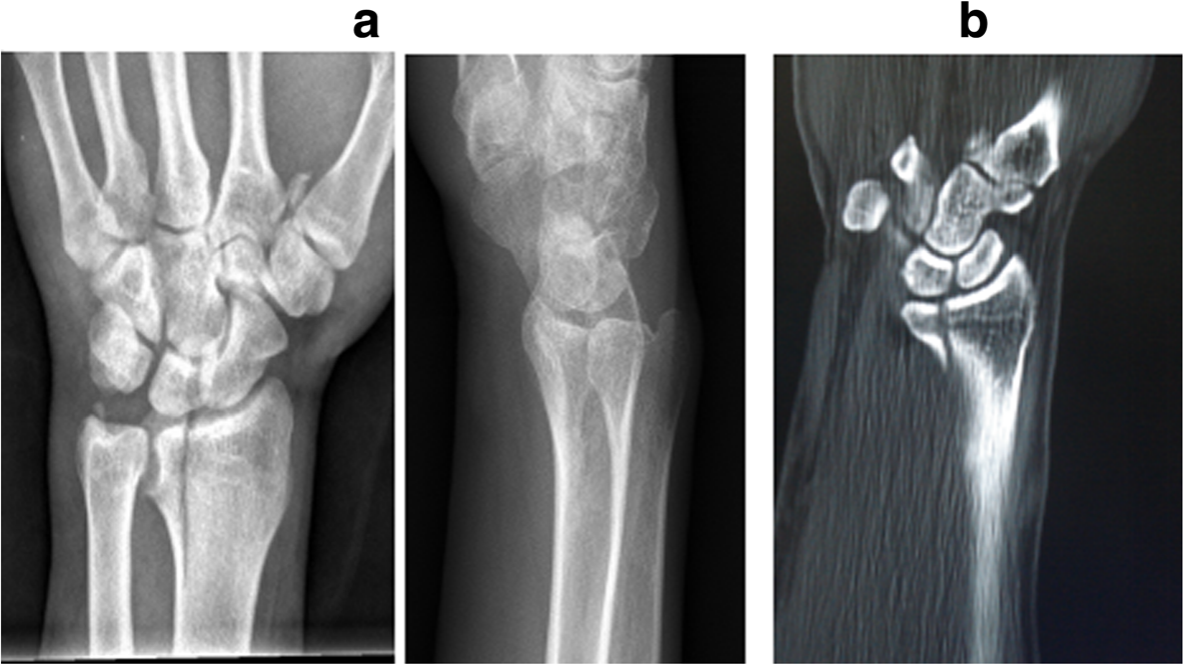
**a**, **b** Split type of ICF

**Fig. 4:**
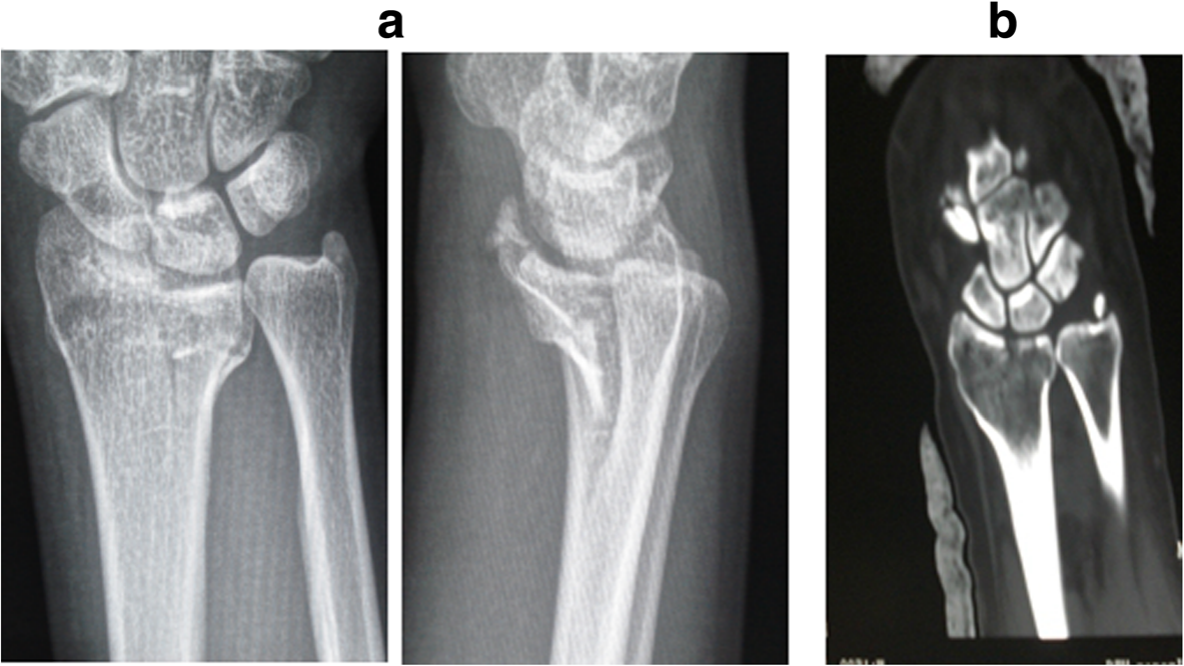
**a**, **b** Collapse type of ICF

**Fig. 5:**
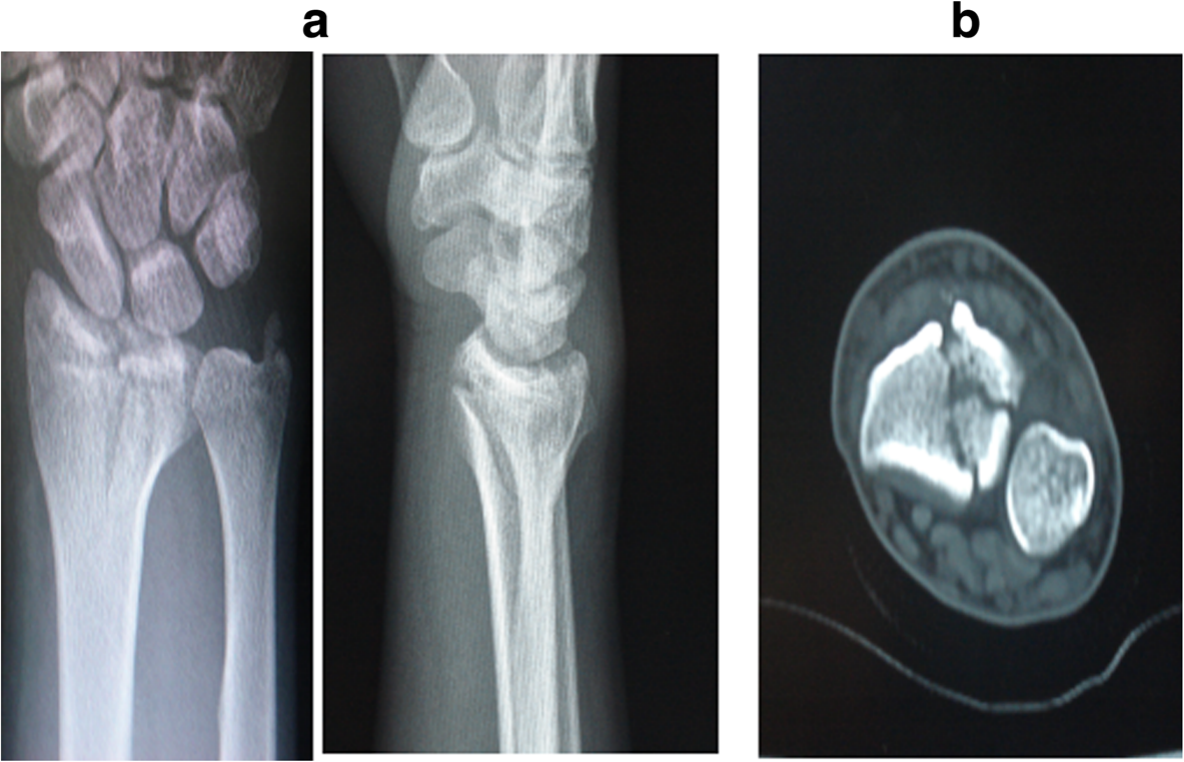
**a**, **b** Collapse with split type of ICF

Based on the site of fractures and the characteristics of morphology, ICF were divided into five types as follows: (1) the volar type is fractures located only in the volar margin of the intermediate column and was without volar margin fractures (Fig. 1); (2) the dorsal type is fractures located only in the dorsal margin of the intermediate column and was without volar margin fractures (Fig. 2); (3) the split type is fractures extending spontaneously to both of the volar and dorsal margins of the intermediate column occurring in the sagittal plane and was often associated with a separation in transverse plane but without significant collapse of the articular surface (Fig. 3); (4) the collapse type is compressive fractures occurring in the center or simultaneously in the volar and dorsal margins of the intermediate column but without obvious separation (Fig. 4); and (5) the split with collapse type is compressive and vertical fractures spontaneously occurring in the volar and dorsal margins of the intermediate column, possibly associated with a separation (Fig. 5). When it was difficult to determine the type of fracture with multiple features, the most severe or obvious feature was used as the basis for assigning the type of fracture.

### Patients

Fractures refer only or mainly to the intra-articular of the middle column of the distal radius were eligible for inclusion. However, fractures were excluded from the study if ICF associated with fractures of the radial articular surface or obvious fractures of the metaphysis of the distal radius.

A total of 74 patients with ICF met the criteria and were included in this study in our hospital from January 2008 to June 2016. The study protocol was approved by the ethics committee of Wuxi the Ninth People’s Hospital Affiliated to Suzhou University. The patients gave their informed consent for the study. There were 45 males and 29 females aged 16~63 years, with an average age of 34.5 years. The cases of fractures were as follows: 26 patients of falls to the ground, 18 patients of a high fall injury, 16 patients of a traffic injury, and 14 patients of impact injury. The information of patients was shown in Table 1. There was an associated separation of the distal radioulnar syndesmosis in 13 patients and associated fractures in other parts in 16 patients. All fractures were identified by standard radiographs and computed tomography scans.

**Table 1:** Patient information and post-operative results

Subject	Volar	Dorsal	Split	Collapse	Collapse with split
Number	16	4	12	28	14
Sex (M/F)	9/7	3/1	8/4	17/11	8/6
Age (year)	18–59	16–61	17–55	18–65	19–62
Distal radioulnar syndesmosis separation	–	–	–	6	7
Displacement (Y/N)	11/5	1/3	10/2	24/4	12/2
Functional recovery (excellent/good/fair/poor)	14/2/0/0	4/0/0/0	11/3/0/0	15/11/2/0	9/4/1/0

### Agreement analysis

Two clinicians were selected as observers to learn the above new classification system. After fully understanding the classification system, they independently classified the fractures in 54 patients with ICF in 2014. After 3 months, the observers re-classified the fractures in 54 patients with ICF. Kappa statistics were used to analyze inter- and intra-observer agreement. The result was interpreted according to convention using the following criteria: < 0 indicated no agreement, 0.00 to 0.02 indicated slight agreement, 0.21 to 0.40 indicated fair agreement, 0.41 to 0.60 indicated moderate agreement, 0.61 to 0.80 indicated substantial agreement, and 0.81 to 1.0 indicated almost perfect agreement.

### Guidelines of the new classification for surgery

Displaced ICF was indicated for surgical treatment. The dorsal type was indicated for the dorsal approach, and the volar and other types were indicated for the volar approach. The collapse type required pry-poking reduction and fixation with screws or bone grafts to support, and the split type required pressurized fixation. These principles guided choices of the surgical approach and reduction-fixation method.

### Treatment method

Fifty-four patients with displaced fractures received surgical treatment. Sixteen patients with non-obvious displaced fractures and four patients with displaced fractures received conservative treatment instead of surgery.

## Results

### Agreement analysis result

The inter- and intra-observer kappa coefficients were 0.875~0.925 and 0.900~0.950, respectively, indicating almost perfect agreement.

### Classification result

The dorsal, volar, split, collapse, and collapse with split types were found in 16, 4, 12, 28, and 14 patients, respectively (Table 1).

### Treatment result

**Fig. 6:**
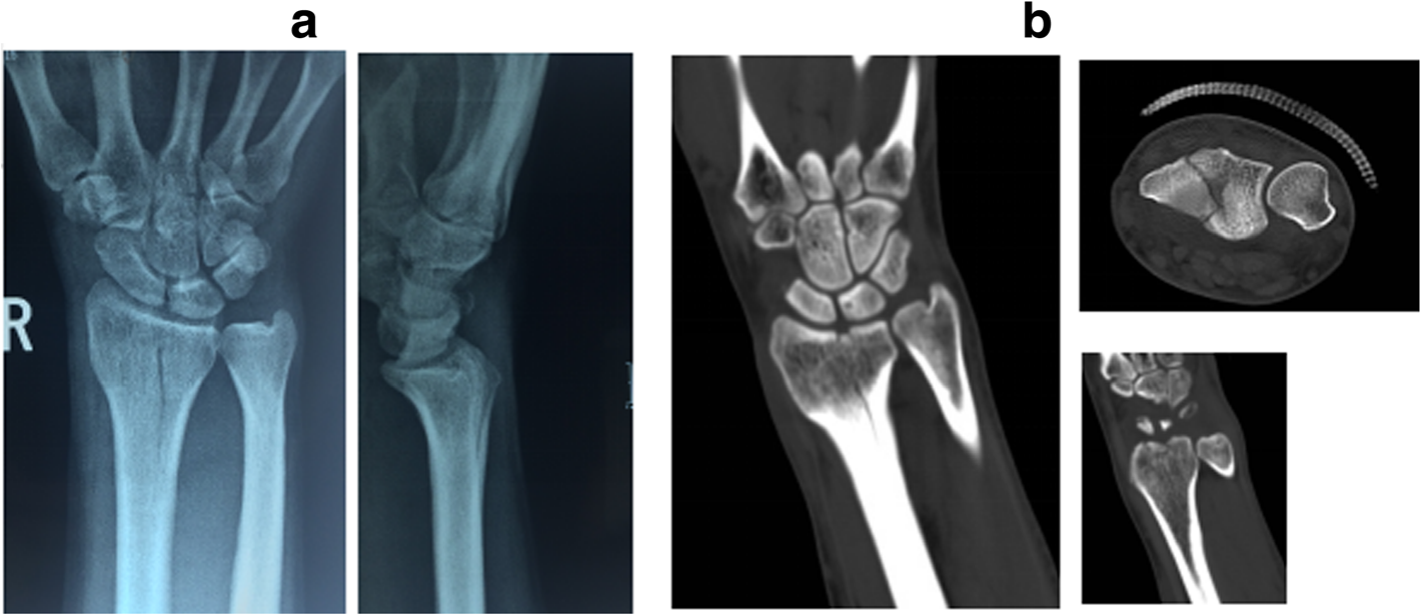
**a**, **b** ICF with collapse accompanied with split type

Seventy-four patients were followed up for 13~ 36 months, with an average of 18.4 months. At the last follow-up, the functional recovery of the wrist was evaluated according to Gartland and Werley score [13], showing excellent in 51 patients, good 20 in patients, and fair in 3 patients (the excellent and good rate was 95.95%). Surgical treatment and outcomes are good as shown in Fig. 6. Among the 3 patients whose functional recovery was rated as fair, 1 belonged to displaced fractures with collapse with split type and had poor reduction; the other 2 belonged to displaced fractures with collapse type but opted for conservative treatment instead of surgery. They had developed traumatic arthritis of the wrist.

## Discussion

Any fracture classification must include all types, reflect the characteristics of fracture, and be of simplicity, vividness, and direct-viewing; otherwise, it is meaningless.

In previous literature, die-punch fractures only referred to the dorsal type, volar type, and collapse type [3–8]. In addition to the dorsal and collapse types, there were also the volar, split, and collapse with split types [9–12]. Different types are closely related to the extent of violent force involved, the local anatomical feature, the difference in bone quality, and the position of the wrist at the time of injury [10]. The peripheral group, consisting of the volar and dorsal types, occurs when the wrist joint is in volar flexion or dorsal extension whileas the central group, consisting of the collapse, split, and collapse with split types, occurs when the wrist joint is in a neutral position as an axial load is passed to the lunate fossa. The normal palmar angulation of the articular surface of the distal radius positions the dorsal articular surface higher than the volar articular surface, as a result, there is usually some degree of dorsal extension of the wrist at the time of injury when the palm strikes the ground during a fall, which more often results in the dorsal type of fractures compared with the volar type [8, 10]. The collapse type of fractures is usually caused by a large violent force or in patients with poor bone quality whileas the split type of fractures is usually caused by a large violent force and occurs in patients with good bone quality. The split type of fracture, due to the fracture separation, is more often accompanied by varying degrees of separation of the distal radioulnar joint. The distal radioulnar volar and dorsal ligaments are the main connecting structures of the distal radioulnar joint that attach to the rim of the sigmoid notch [14]. When the peripheral group fractures involve the distal radioulnar volar or dorsal ligaments, they often cause separation of the distal radioulnar joint.

According to the new classification system, 74 patients with ICF were as follows: the collapse type in 28 patients, the dorsal type in 16 patients, the collapse with split type in 14 patients, the split type in 12 patients, and the volar type in 4 patients; 13 patients who had an associated separation of the distal radioulnar joint were as follows: 6 patients were the split type and 7 patients were the collapse with split type. The type distribution and associated injury in the 74 patients coincide well with the characteristics of the ICF; therefore, the new classification system better reflects the characteristics of the violence of force, local anatomy, bone quality of the patient, and the wrist position at the time of injury. In addition, it has characteristics of simplicity, vividness, and direct-viewing.

**Fig. 7:**
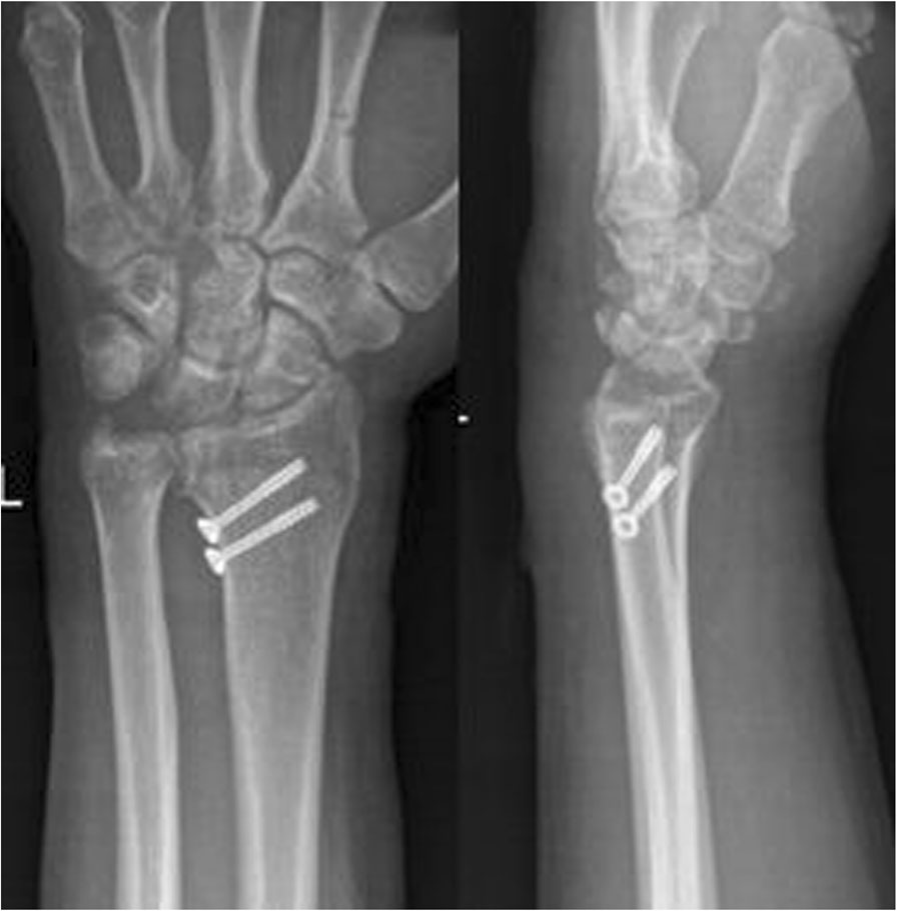
Post-operative X-ray film of ICF

One of the main purposes of any classification system is to create a common language for individuals who treat fractures, thereby promoting efficient and reliable communication. Achieving this level of communication requires a minimum degree of inter-observer and intra-observer disagreement. In this study, it was difficult to categorize/classify fractures in seldom patients with a slight fracture (Fig. 7). The inter-observer kappa coefficient was 0.875~0.925, and the intra-observer kappa coefficient was 0.900~0.950, which means as a whole the agreement of the new classification system was good.

Another main purpose of any classification system is to assist in clinical decision-making with respect to the need for operative versus non-operative care, the surgical approach, the reduction-fixation method, and outcome prediction. Currently, most authors select surgical treatment for displacement of more than 1~2 mm for intra-articular fractures of the distal radius [15–21]. Therefore, we regarded displaced ICF as unstable fracture for which surgical treatment was indicated, and non-obvious displaced ICF as stable fracture for which conservative treatment was indicated. Surgical approach must be selected according to the position of the fracture, a dorsal approach was used for the dorsal type, a volar approach was used for the volar type, the central group may use a dorsal or a volar approach, but the volar approach is superior to the dorsal approach as it may avoid injury to the tendons. The collapse type requires pry-poking reduction and fixation with screws or bone grafts to support the collapsed fractures, and the split type requires pressurized fixation as separation of fractures. The above principles are usually accepted for intra-articular fractures of the distal radius [14, 18, 20–22]. Under the guideline of the new classification, surgery was performed in 54 cases with displaced fractures. In the surgery group, only 1 patient with displaced collapse with split type developed traumatic arthritis of the wrist as poor fracture reduction and had a fair functional recovery; the excellent and good rate in the surgery group was 95.95%, which is greater than or equal to the average of reported outcomes of intra-articular and unstable fractures of the distal radius [14, 16–22].

## Conclusions

In the conservative group, 2 of the 4 patients with displaced collapse type as declining surgery developed traumatic arthritis of the wrist and had a fair functional recovery. The results indicated that the collapse and collapse with split types are the most severe types, which may lead to traumatic arthritis of the wrist and affect functional recovery if not rightly treated [22]. Under the guidelines of the new classification for surgery, the outcome was quite satisfactory, suggesting that the treatment method and prognoses were diverse for different types of ICF. Therefore, the new classification for ICF could serve as an important reference for treatment decision-making and evaluation of prognosis.

## Abbreviations

ICF: Intermediate column fractures of the distal radius
